# Plasma *AR* Copy Number Changes and Outcome to Abiraterone and Enzalutamide

**DOI:** 10.3389/fonc.2020.567809

**Published:** 2020-09-24

**Authors:** Giorgia Gurioli, Vincenza Conteduca, Cristian Lolli, Giuseppe Schepisi, Stefania Gargiulo, Amelia Altavilla, Chiara Casadei, Emanuela Scarpi, Ugo De Giorgi

**Affiliations:** ^1^Biosciences Laboratory, Istituto Scientifico Romagnolo per lo Studio e la Cura dei Tumori (IRST) IRCCS, Meldola, Italy; ^2^Department of Medical Oncology, Istituto Scientifico Romagnolo per lo Studio e la Cura dei Tumori (IRST) IRCCS, Meldola, Italy; ^3^Unit of Biostatistics and Clinical Trials, Istituto Scientifico Romagnolo per lo Studio e la Cura dei Tumori (IRST) IRCCS, Meldola, Italy

**Keywords:** cell free DNA, androgen receptor, copy number changes, abiraterone acetate, enzalutamide, clinical outcome

## Abstract

**Introduction:** Plasma androgen receptor (*AR*) copy number (CN) status identifies castration-resistant prostate cancer (CRPC) patients with worse outcome on abiraterone/enzalutamide. However, the impact of *AR* CN changes on clinical outcome in CRPC is unknown.

**Materials and Methods:** Plasma samples from 73 patients treated with abiraterone or enzalutamide were collected at baseline and at the time of progression disease (PD). Droplet digital polymerase chain reaction was used to assess *AR* CN status.

**Results:** We showed that 11 patients (15.1%) changed *AR* CN status from baseline to PD (9 patients from normal to gain, 2 from gain to normal). Patients changing *AR* CN status from normal at baseline to gain at PD had intermediate median overall survival (OS) of 20.5 months (95% CI = 8.0–44.2) between those who remained *AR* CN normal from baseline to PD (27.3 months [95% CI = 21.9–34.4]) and those who remained *AR* CN gain from baseline to PD (9.1 months [95% CI = 3.8–14.5], *p* < 0.0001). Patients changing *AR* CN from normal at baseline to gain at PD had a median progression-free survival (PFS) of 9.2 months (95% CI = 2.0–14.7), patients who remained *AR* CN normal had a median PFS of 9.1 months (95% CI = 7.2–10.1), and patients who remained *AR* CN gain had a median PFS of 5.4 (95% CI = 3.6–6.5, *p* = 0.0005). Both OS and PFS were not significantly different between patients with *AR* CN that changes from normal to gain and patients with stable *AR* CN normal.

**Conclusions:** We showed that CRPC patients changing *AR* CN status from baseline to progression time point had intermediate OS and we suggested that *AR* CN evaluation at baseline could be the most informative for clinical outcome of CRPC patients treated with abiraterone or enzalutamide. Larger prospective studies are warranted.

## Introduction

Abiraterone acetate and enzalutamide belong to therapies introduced in the past years for patients with metastatic castration-resistant prostate cancer (CRPC), improving patient survival and quality of life (1–4). During therapy, the fractions of DNA alterations may vary under treatment selection, giving rise to alterations originally present in a very small number of cancer cells (5). Moreover, currently there is not yet a validated sequence of therapies and patient selection strategies, so there is now an urgent need to identify noninvasive biomarkers able to guide treatment selection for CRPC personalized medicine. Cell free DNA (cfDNA) has emerged as a minimally invasive and good source of biomarkers deriving from multiple metastases, suggesting its role in monitoring clinical outcome and tumor heterogeneity (6–8). Literature data reported that changes in cfDNA concentration correlate with radiological progression-free survival (PFS) and overall survival (OS) and may be used as independent prognostic biomarker of response to taxanes (9).

Androgen receptor (AR) has a key role in prostate cancer development and progression (10, 11). Copy number (CN) of *AR* has been well-investigated and is considered to be one of the main mechanisms of hormone-sensitive to hormone-resistant transition (12). Plasma *AR* CN at baseline of abiraterone and enzalutamide was associated with resistance to these therapies, both in pre- and post-chemotherapy with docetaxel (13). In this study, we evaluate the role of plasma *AR* CN changes on clinical outcome in CRPC patients treated with abiraterone or enzalutamide.

## Materials and Methods

### Patients

From August 2012 to June 2016, CRPC patients with histologically confirmed diagnosis of prostate adenocarcinoma were enrolled. Patients were treated with abiraterone or enzalutamide in pre- or post-chemotherapy settings. Treatment was continued until evidence of disease progression or unacceptable toxicity. The choice of therapy was at the discretion of the treating physician.

Serum prostate-specific antigen (PSA), alkaline phosphatase (ALP), serum lactate dehydrogenase (LDH), cell blood count to determine neutrophil to lymphocyte ratio were measured within 1 week of starting therapy and once per month thereafter. Radiographic disease was assessed with computed tomography (CT) and bone scans at the time of screening and once every 12 weeks during treatment. ^18^F-fluorocholine positron emission tomography/computed tomography (FCH-PET/CT) was performed after 12 ± 4 weeks of treatment.

Progression disease (PD) was assessed considering radiographic evidence of new lesions by bone scintigraphy and/or new or enlarging soft tissue lesions by CT or magnetic resonance imaging, per the Prostate Cancer Clinical Trials Working Group 3 guidelines (14). The study was approved by the Local Ethics Committee and informed consent was obtained from each patient for their biological material to be used for research purposes (CEIIAV IRSTB048).

### Molecular Analysis

Peripheral blood samples were collected before initiating therapy with abiraterone or enzalutamide and at the time of PD. The blood was drawn into 10 ml ethylene diamine tetra-acetic acid (EDTA) tubes, maintained at room temperature, processed within 30 min and stored at −80°C. Circulating DNA was extracted from 1 or 2 ml of plasma using QIAamp Circulating Nucleic Acid Kit (Qiagen). Total extracted DNA was quantified with the Quant-iT high sensitivity PicoGreen double-stranded DNA Assay Kit (Invitrogen). We performed plasma *AR* CN analysis with a multiplex digital droplet polymerase chain reaction (Biorad) assay using three reference genes: *NSUN3, ElF2C1*, and *AP3B1* and *ZXDB* at Xp11.21 as a control gene not involving the whole arm of the chromosome, as previously described (15).

### Statistical Analyses

PFS was defined as the time elapsed between the date of start of therapy and the date of radiological/clinical or biochemical progression or last tumor evaluation. OS was defined as the time elapsed between the date of start of therapy and the date of death from any cause or the date of last follow-up. PFS and OS were estimated using Kaplan–Meier method and compared using logrank test. *P-*values were two-sided and a *p* < 0.05 was considered as statistically significant. Statistical analyses were performed with SAS statistical software, version 9.4 (SAS Institute, Cary, NC, USA).

## Results

**Figure 1:**
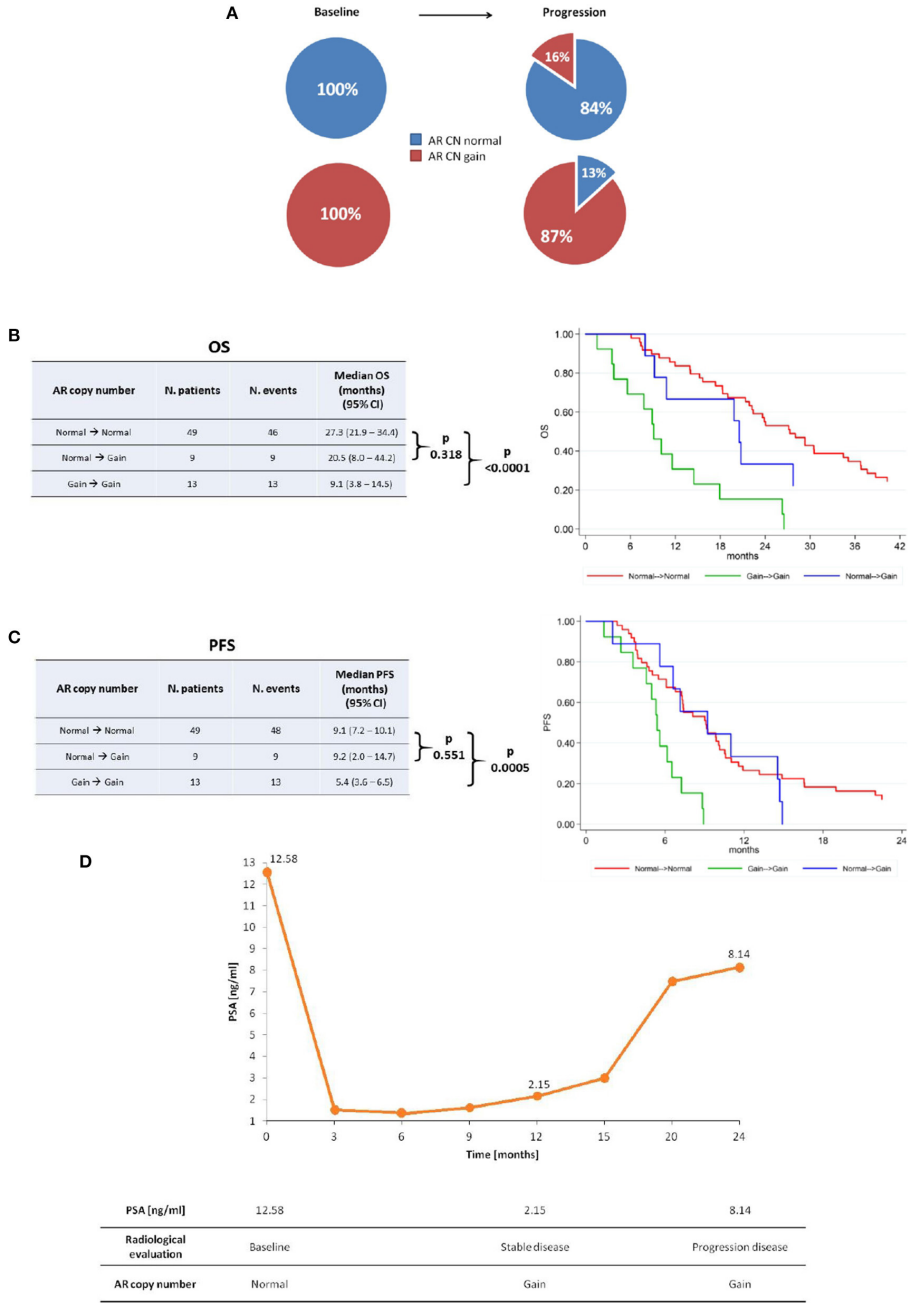
*AR* copy number changes. **(A)** *AR* copy number changes from baseline to progression time point. Median OS **(B)** and PFS **(C)** of stable *AR* CN normal, *AR* CN changing from normal to gain, and stable *AR* CN gain patients with the corresponding Kaplan–Meier curves. **(D)** Representative case that changed *AR* CN from normal to gain at stable disease and remained gain at disease progression, even though PSA response occurred.

This study evaluated 73 patients with sufficient plasma DNA for *AR* CN detection in both baseline and progression samples. Of these, 35 (48%) and 38 (52%) received abiraterone or enzalutamide pre- and post- chemotherapy, respectively. When comparing the baseline patient characteristics according to therapy, post-chemotherapy setting displayed a higher Gleason score, a greater incidence of bone and visceral metastases as well as higher levels of ALP and LDH (as shown in Table 1). We found that 84% (49) of 58 patients with *AR* CN normal and 87% (13) of 15 with *AR* CN gain at baseline showed no changes in *AR* CN status in their PD sample (Figure 1A), observing a 15.1% conversion rate of *AR* CN status from baseline to PD time point. Clinical outcome in terms of OS (Figure 1B) and PFS (Figure 1C) was evaluated. In univariate analyses, we found that patients who were *AR* CN normal at baseline, then converted to *AR* CN gain at PD, had intermediate OS between those who were *AR* CN normal at baseline and remained normal at PD and those who were *AR* CN gain at baseline and remained gain. Patients changing *AR* CN from gain to normal were not included in the analyses because the group is only represented by two men. Particularly, we showed that patients changing *AR* CN from normal to gain had an intermediate median OS of 20.5 months (95% CI = 8.0–44.2) between patients with stable *AR* CN normal that presented a median OS of 27.3 months (95% CI = 21.9–34.4) and patients with stable *AR* CN gain with a median OS of 9.1 (95% CI = 3.8–14.5, *p* < 0.0001). The two patients changing *AR* CN from gain to normal presented an OS of 8.5 and 17.4 months, respectively. No significantly worse OS was observed for patients changing *AR* CN from normal to gain compared to patients with stable *AR* CN normal (*p* = 0.318, Figure 1B).

**Table 1:** Patient characteristics.

	**Abi/Enza pre-docetaxel (*n* = 35)**	**Abi/Enza post-docetaxel (*n* = 38)**	
	***N* (%)**	***N* (%)**	***p***
**Age**, years: median value (IQR)	72 (68–80)	75 (72–77)	0.557
**Gleason score**			
6–7	18 (52.9)	8 (25.8)	
8–10	16 (47.1)	23 (74.2)	0.027
Unknown/missing	1	7	
**Metastastic sites**			
Bone	18 (51.4)	34 (89.5)	0.0004
Visceral	2 (5.7)	12 (31.6)	0.005
Liver	1 (2.9)	3 (9.4)	0.342
Nodal	16 (45.7)	17 (44.7)	0.934
**Previous abi or enza treatment**			
No	35 (100)	28 (73.7)	
Yes	0	10 (26.3)	–
**Previous cabazitaxel treatment**			
No	35 (100)	35 (92.1)	
Yes	0	3 (7.9)	0.241
***AR*** **copy number**			
Normal	29 (82.9)	29 (76.3)	
Gain	6 (17.1)	9 (23.7)	0.492
**Baseline ALP**, U/L: median value (IQR)	88 (67–121)	109 (79–196)	0.129
<129	28 (80.0)	22 (57.9)	
≥129	7 (20.0)	16 (42.1)	0.044
**Baseline LDH**, U/L: median value (IQR)	163 (143–190)	179 (115–968)	0.047
<225	32 (91.4)	32 (84.2)	
≥225	3 (8.6)	6 (15.8)	0.482
**Baseline NLR:** median value (IQR)	2.49 (2.04–3.31)	2.41 (1.86–4.16)	0.904
<3	22 (62.9)	21 (55.3)	
≥3	13 (37.1)	17 (44.7)	0.513
**Baseline Neutrophil:** median value (IQR)	3,560 (3,090–5,090)	3,590 (2,850–4,960)	0.904
**Baseline Lymphocyte:** median value (IQR)	1,540 (1,090–1,761)	1,310 (1,050–1,740)	0.202
**Baseline PSA**, ng/mL: median value (IQR)	32.13 (6.80–68.38)	65.06 (19.76–182.10)	0.129

*IQR, interquartile range; abi, abiraterone; enza, enzalutamide; AR, androgen receptor; ALP, alkaline phosphatase; LDH, lactate dehydrogenase; NLR, neutrophil to lymphocyte ratio; PSA, prostate specific antigen*.

Patients changing *AR* CN from normal to gain status had a median PFS of 9.2 months (95% CI = 2.0–14.7), patients with stable *AR* CN normal presented a median PFS of 9.1 months (95% CI = 7.2–10.1) and patients with stable *AR* CN gain had a median PFS of 5.4 (95% CI = 3.6–6.5, *p* = 0.0005). The two patients changing *AR* CN from gain to normal presented a PFS of 8.5 and 3.8 months, respectively. No significantly worse PFS was observed for patients changing *AR* CN from normal to gain compared to patients with stable *AR* CN normal (*p* = 0.551) (Figure 1C).

Correlation analysis between *AR* CN changes and PSA change was performed and we did not found a statistically significant correlation, as shown in Supplementary Table 1. PSA change was defined as a PSA decline >50% from baseline at first radiological evaluation (after about 3 months from starting therapy) according to the Prostate Cancer Clinical Trials Working Group 3 guidelines. PSA decline was available for 66 patients and we did not consider the two patients changing *AR* CN from gain to normal. Interestingly, we found that all patients changing *AR* CN status from normal to gain (8) did not show PSA decline.

## Discussion

This study represents the first evidence of the impact of plasma *AR* CN changes from baseline to progression time point on clinical outcome of CRPC patients. We identified a low conversion rate of *AR* CN status, suggesting that, for most of patients, *AR* CN does not change under the pressure of abiraterone and enzalutamide. These results confirm those found by Romanel et al. (13) that showed that *AR* CN status in individual metastases does not noticeably change with treatment, but the impact on clinical outcome was not investigated.

We hypothesized that this could be challenging for physicians in helping treatment decision and possibly substituting therapy if *AR* CN change occurs during treatment, minimizing overtreatment. Our results showed that patients changing *AR* CN from normal to gain had intermediate median OS between patients with stable *AR* CN normal and patients with stable *AR* CN gain. This trend of intermediate prognosis was not observed for PFS, probably because *AR* CN was analyzed at the time of disease progression, impeding to impact on PFS itself. However, since the number of patients changing *AR* CN from normal to gain (9) was very low, additional validation is needed. Similarly, only 2 of the 15 *AR* CN gain patients became normal at PD, so it does not allow to identify the role of *AR* CN conversion in this setting of patients. Moreover, since both PFS and OS were not significantly different between patients with *AR* CN that changes from normal to gain and patients with stable *AR* CN normal, we hypothesized that the difference on clinical outcome depends on *AR* CN status at baseline. For this reason, we suggested that baseline assessment could be the most relevant time point to consider for *AR* CN status in therapeutic decision making.

Clinical utility of cfDNA biomarkers has emerged because of the impracticality of sampling bone metastatic tissue in CRPC patients. Circulating DNA is easy to obtain for serial monitoring of tumor dynamics, allowing the recognition of tumor heterogeneity (15). Primary and acquired resistance to AR-targeted therapies has been associated with mutations or amplification of *AR* gene (8, 16–18) and the expression of AR splice variants (i.e., AR-V7) in circulating tumor cells (CTC) or in metastatic tissues (19, 20). Hörnberg et al. (20) found that high levels expression of AR-V7 in prostate cancer bone metastases correlates with particularly poor prognosis. Antonarakis et al. (21) showed that 14% of patients AR-V7 negative at their baseline CTC samples converted to AR-V7 positive during the course of treatment with abiraterone/enzalutamide or at the time of PD and demonstrated that these patients had intermediate clinical outcomes (PSA response rates, PFS) between AR-V7 negative patients that remained negative and AR-V7 positive patients who remained all positive (21). Changes in AR-V7 status (i.e., conversion from AR-V7 negative to positive during AR-targeted therapies and reversion from AR-V7 positive to negative during taxanes) highlight the potential role of AR-V7 as a dynamic marker (22). However, large cohorts studies are needed to better clarify the predictive ability of AR-V7, standardize sensitive, and cost-sustainable clinical laboratory assays for the measurement of patients AR variants (23).

Actually, it is unclear whether lethal phenotype derives from multiple foci with different genomic patterns that metastasize or it depends on a single clone that maintains dominance during the course of the disease. These mechanisms could select specific clones, shedding light on the biological processes underlying the conversion of *AR* CN status (from normal to gain or from gain to normal) during therapies (5).

In treatment-naïve patients, intra-tumoral heterogeneity increases as the tumor burden increases, and individual metastatic lesions are affected by their local microenvironment. CRPC cells modify themselves because of the presence of distinct clones selected by therapies, that could be a basis of treatment resistance (7, 24). Abiraterone acetate and enzalutamide were introduced into clinical practice to overcome AR signaling reactivation after androgen deprivation therapies (25, 26). Since treatment resistance mechanisms occurs, there is an urgent need to identify the predictive and prognostic biomarkers for treatment selection in CRPC.

The present study represents the first evidence of the impact of plasma *AR* CN changes during abiraterone and enzalutamide treatments. We showed that conversion rate of *AR* CN was low and that CRPC patients changing *AR* CN status from baseline to progression time point had intermediate OS. Moreover, we suggested that *AR* CN evaluation at baseline could be the most informative for clinical outcome of CRPC patients treated with abiraterone or enzalutamide. The major limitations of the study included the relatively small number of patients enrolled and the retrospective nature of the study. Moreover, we evaluated *AR* CN status at baseline and progression time points, but *AR* CN at first radiological evaluation after 3 months of therapy is needed, as it represents the most clinically significant time point, according to Prostate Cancer Clinical Trials Working Group 3 guidelines. Finally, we did not distinguished circulating tumor DNA (ctDNA) from normal DNA present in cfDNA that potentially affects the detection of *AR* gain in patients with a low proportion of ctDNA. Larger prospective biomarkers study is warranted.

## Data Availability Statement

The raw data supporting the conclusions of this article will be made available by the authors, without undue reservation.

## Ethics Statement

The studies involving human participants were reviewed and approved by COMITATO ETICO della Romagna. The patients/participants provided their written informed consent to participate in this study.

## Author Contributions

Conceptualization: GG and VC. Methodology, writing—original draft preparation, and writing—review and editing: GG. Software: SG. Validation: UD, VC, and ES. Formal analysis: ES. Investigation: CL. Resources: GS. Data curation: AA. Visualization: CC. Supervision: UD. Project administration: VC. All authors have read and agreed to the published version of the manuscript.

## Conflict of Interest

GG has received travel support from Sanofi. VC has received speaker honoraria or travel support from Astellas, Janssen-Cilag, and Sanofi-Aventis, and has received consulting fee from Bayer. CL has received honoraria for consulting (advisory board) from Bristol-Myers Squibb and Janssen-Cilag. UD has served as consultant/advisory board member for Astellas, Bayer, BMS, Ipsen, Janssen, Merck, Pfizer, Sanofi, and has received travel support from BMS, Ipsen, Janssen, Pfizer, and has received research funding from AstraZeneca, Roche, Sanofi (Inst). The remaining authors declare that the research was conducted in the absence of any commercial or financial relationships that could be construed as a potential conflict of interest. The reviewer FM declared past co-authorships with the authors to the handling editor.

## Abbreviations

AR: androgen receptor
CN: copy number
CRPC: castration-resistant prostate cancer
PD: progression disease
OS: overall survival
PFS: progression-free survival
cfDNA: Cell free DNA
PSA: prostate-specific antigen
ALP: alkaline phosphatase
LDH: lactate dehydrogenase
CT: computed tomography
AR-V7: AR splice variant 7
CTC: circulating tumor cells
ctDNA: circulating tumor DNA.
