# Supplementary material for: Plasma AR Copy Number Changes and Outcome to Abiraterone and Enzalutamide
Source: Front Oncol. 2020 Sep 24;10:567809. doi: 10.3389/fonc.2020.567809 (PMC7542981; doi:10.3389/fonc.2020.567809)
Supplement: Supplementary file 1 [file Table_1.docx]

**Table S1.** *AR* CN changes and PSA change correlation

|  | **PSA change (decline)** | | |
| --- | --- | --- | --- |
|  | **No** | **Yes** |  |
|  | **N (%)** | **N (%)** | **p value** |
| **AR CN changes** |  |  |  |
| **No** | 47 (85.5) | 11 (100) |  |
| **Yes** | 8 (14.5) | 0 | 0.334 |
